# Research and Remedies

**Published:** 1913-07-19

**Authors:** 


### RESEARCH AND REMEDIES.

### The Ætiology of Chorea.

At a recent meeting of the Société Médicale des Hôpitaux Dr. Milian gave an account of some work he had of late been engaged in with regard to the ætiology of chorea. In fifteen cases of the disease, eleven were found to be suffering from undoubted syphilis as shown by a positive Wassermann reaction, two were probably syphilitic and two doubtfully so. The author had previously reported to the same Society two cases of chorea with undoubted syphilis, and he mentioned two others previously reported by Apert and Rizat. In the present series a history of rheumatism could be obtained in three cases only, of which two were syphilitic, so that the articular and muscular pains might have been of a syphilitic nature. The good effects of arsenic in chorea lend support to the hypothesis of the syphilitic origin of chorea. In the discussion which followed, the author met with but little support for his theory. M. Babboneix said that out of forty-five cases seen by him thirty-six were suffering from hereditary syphilis, so that syphilis may have a predisposing or determining influence in chorea, but he declined to generalise on the subject. Other speakers directly negatived the hypothesis as being against clinical and *post-mortem* experience.

### Treatment of Anthrax by Salvarsan.

Anthrax would seem to be on the increase in Germany if the number of articles which have lately been published on this subject in the German medical press can be taken as a criterion. Some encouraging results in such cases have followed the use of salvarsan. Bettmann and Laubenheimer, in the *Deutsche Med. Woch.*, report two cases cured after two injections of the drug. In guinea-pigs they found that the anthrax bacilli disappeared from the blood and tissues, if salvarsan were injected at periods varying from twenty minutes to six hours after inoculation with the bacilli. No therapeutic effect could, however, be obtained if the injections were delayed until sixteen to twenty-two hours after inoculation. Buker, in the *Deutsche Zeitsch. f. Chirurgie*, mentions a case of very grave prognosis which recovered after an intravenous injection of the drug. In the *Münch. Med. Woch.* Schuster has shown that a fatal issue can be prevented in inoculated rabbits by injections of salvarsan calculated at 0.04 gram of the drug per kilogram of body weight of the animal. The drug acts merely as a disinfectant, for it confers no immunity, nor can antibodies be found in the blood.

### A New Remedy for Ringworm.

Dr. Agnes Savill speaks very highly of a lotion, recommended some time ago by Dr. Winkelried Williams, containing seven grains of picric acid and half an ounce of camphor in half an ounce of rectified spirit, as a depilatory and germicide for ringworm. The hair is cut short round the diseased patch in the usual way, and the lotion painted on with a camel-hair brush morning and evening. As the lotion evaporates a yellow powder accumulates on the head. This powder is to be washed away at least twice a week, and it is also important that the hair should be cut short by clipping or shaving two or three times a week. When all these details are carefully observed the hair becomes loosened in about three weeks and can be easily epilated. The results are described as exceedingly satisfactory.

### Eserine and Epilepsy.

The fact that eserine is capable of unfavourably influencing epilepsy is probably unfamiliar to 99 per cent. of the profession, although a statement to this effect appears in Dr. Hale White's admirable and popular text-book of pharmacology. A very curious case, which illustrates the connection between epilepsy and eserine, is narrated in the *Ophthalmoscope* by Dr. T. M. Bride. A female patient was admitted to the Royal Eye Hospital, Manchester, for chronic glaucoma, and was treated by eye-drops of 1-per-cent. eserine six times a day. She was forty-six years of age—long past the usual age for the onset of epilepsy—and had never been epileptic, though some of her relatives had suffered from that disease. Eight days after her admission she had four epileptic fits. The eserine was stopped, but on the following day a status epilepticus supervened, and for some hours her condition was critical; speedy recovery then ensued. Twelve days after the onset of the fits eserine was once more inadvertently applied to the eye: twelve hours later two epileptic fits occurred. After this pilocarpine was used for the eyes instead of eserine, and no more fits have been reported. The case is remarkable, and bears lessons very plainly to be recognised.

